# Neurodevelopmental outcome of extremely low birth weight infants at 24 months corrected age: a comparison between Griffiths and Bayley Scales

**DOI:** 10.1186/s12887-015-0457-x

**Published:** 2015-09-30

**Authors:** Odoardo Picciolini, Chiara Squarza, Camilla Fontana, Maria Lorella Giannì, Ivan Cortinovis, Silvana Gangi, Laura Gardon, Gisella Presezzi, Monica Fumagalli, Fabio Mosca

**Affiliations:** NICU, Department of Clinical Sciences and Community Health, Fondazione IRCCS Ca’ Granda Ospedale Maggiore Policlinico, Università degli Studi di Milano, Via Della Commenda 12, Milan, 20122 Italy; Department of Clinical Sciences and Community Health-Laboratory of Medical Statistics, Biometry and Epidemiology, Università degli Studi di Milano, Via Della Commenda 12, Milan, 20122 Italy

**Keywords:** Bayley-II, Bayley-III, Griffiths, Developmental assessment, Extremely low birth weight infants

## Abstract

**Background:**

The availability of accurate assessment tools for the early detection of infants at risk for adverse neurodevelopmental outcomes is a major issue. The purpose of this study is to compare the outcomes of the Bayley Scales (Bayley-II vs Bayley-III) in a cohort of extremely low birth weight infants at 24 months corrected age, to define which edition shows the highest agreement with the Griffiths Mental Development Scales Revised.

**Methods:**

We performed a single-centre cohort study. We prospectively enrolled infants with a birth weight of 401–1000 g and/or gestational age < 28 weeks. Exclusion criteria were the presence of neurosensory disabilities and/or genetic abnormalities. Infants underwent neurodevelopmental evaluation at 24 months corrected age using the Griffiths and either the Bayley-II (birth years 2003–2006) or the Bayley-III (birth years 2007–2010).

**Results:**

A total of 194 infants were enrolled. Concordance was excellent between the Griffiths and the Bayley-III composite scores for both cognitive language and motor abilities (weighted *K* = 0.80 and 0.81, respectively) but poorer for the Bayley-II (weighted *K* = 0.63 and 0.50, respectively). The Youden’s Index revealed higher values for the Bayley-III than for the Bayley-II (75.9 vs 69.6 %). Compared with the Griffiths, the Bayley-III found 3 % fewer infants as being severely impaired in cognitive-language abilities and 7.8 % fewer infants as being mildly impaired in motor skills while the Bayley-II showed, compared with the Griffiths, higher rates of severely impaired children both for cognitive-language and motor abilities (14.1 and 15.3 % more infants respectively).

**Discussion:**

Our study suggests that the Bayley-III, although having a higher agreement with the Griffiths compared to the Bayley-II, slightly tends to underestimate neurodevelopmental impairment compared with the Griffiths, whereas the Bayley-II tends to overestimate it.

**Conclusions:**

On the basis of these findings, we recommend the use of multiple measures to assess neurodevelopmental outcomes of extremely low birth weight infants at 24 months.

## Background

Survival of extremely low birth weight (ELBW) infants has dramatically increased in recent decades because of advances in perinatal and neonatal care [1, 2]. However, rates of disability, especially at the lowest gestational ages, remain high [3]. As a consequence, the availability of accurate developmental assessments for the early detection of infants at high risk of adverse neurodevelopmental outcomes has become a major issue. Indeed, early confirmation of developmental impairment is important so that early referral for intervention can be made to maximise children’s abilities and to assist in their transition to school.

The Bayley Scales are widely applied to identify infants with or at risk for developmental impairment, both in clinical and research settings [4, 5]. The first two editions of the scales [6, 7] yielded only a Mental Development Index (MDI) and a Psychomotor Development Index (PDI). The revised structure of the Bayley-III [8], which includes distinct composite scores (Cognitive, Language and Motor), allows a more precise assessment of specific developmental domains. Nevertheless, clinicians have consistently found that Bayley-III composite scores are up to 10 points higher than those of Bayley-II [9, 10]. Thus, concerns have arisen that the Bayley-III may underestimate developmental impairment in clinical groups [11], reducing the number of children eligible for early intervention programmes.

Up to now, few studies have addressed the agreement between the Bayley Scales outcomes and other valid and reliable standardized developmental instruments on the same study group.

The Griffiths Mental Development Scales [12] are a widely used developmental assessment procedure, showing continuing validity over time and across cultures [13–15]. They were first published in 1970 and underwent a re-standardization in 1996 for the 0–2 years version [12, 16].

The Griffiths General Quotient at 2 and 3 years of age has been found to strongly correlate with intellectual ability at 5 years on the Stanford Binet [17] and moderately with the Wechsler Preschool and Primary Scale for Intelligence-Revised (WPPSI-R) [18]. McMichael [19] assessed low-birthweight infants at 1 and 3 years on the Griffiths and at 24 months on the Bayley-III, and found that the Bayley-III composite scores were almost a standard deviation higher than those on the Griffiths at both 12 and 36 months.

The aim of this study was to evaluate the developmental outcomes of a cohort of extremely low birth weight infants assessed at 24 months corrected age using both the Bayley Scales II and III and the Griffiths, so as to define which edition of the Bayley Scales better agrees with the Griffiths. The null hypothesis to be tested was that the agreement between the Griffiths and the Bayley-III would not be higher than the agreement between the Griffiths and the Bayley-II.

## Methods

### Study design and participants

We performed a single-centre longitudinal cohort study. The study was approved by the Ethics Committee of the Fondazione IRCCS Ca’ Granda Ospedale Maggiore Policlinico and written informed consent was obtained from all parents.

Inclusion criteria were having a birth weight between 401 and 1000 g at birth (ELBW) and/or being born between 22 and 27^+6^ weeks gestation (extremely low gestational age newborns: ELGAN). Exclusion criteria were the presence of neurosensory disabilities (blindness, deafness) and/or genetic abnormalities.

**Fig. 1:**
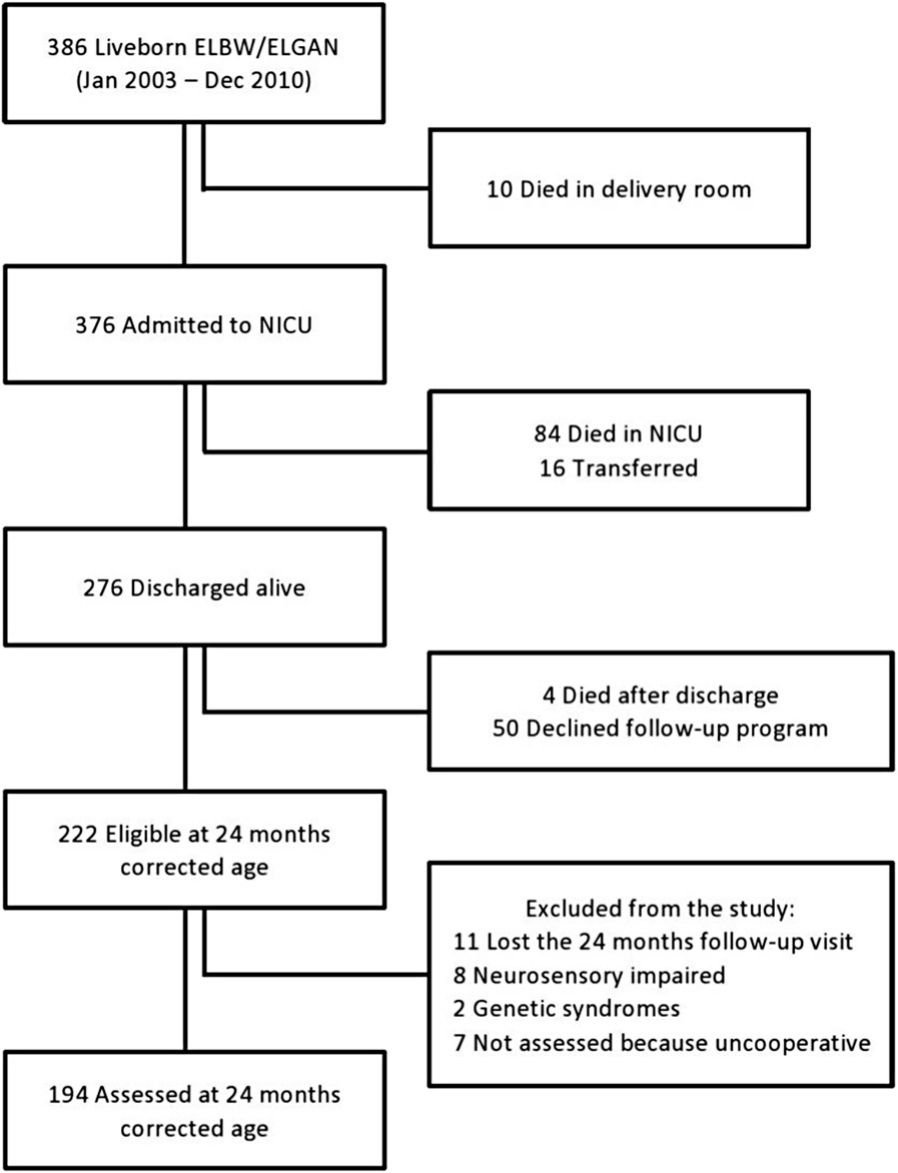
Flow chart of the study

The flow chart of the study is shown in Fig. 1. Of all the 376 consecutive infants admitted to NICU Fondazione IRCCS Ca’ Granda Ospedale Maggiore Policlinico between 2003 and 2010, 276 (73 %) were discharged home alive. Of these, 222 (80 %) returned for the 24 months corrected age follow-up visit and 194 (70 %) infants entered the study.

All infants participating in the study were registered in the Vermont Oxford Network [20] and were scheduled to be prospectively followed up to 24 months corrected age.

The infants were divided into two groups according to the study period: Group 1 (*N* = 92) infants born between 2003 and 2006, and Group 2 (*N* = 102) infants born between 2007 and 2010.

Basic subjects’ characteristics (sex, birth weight, being adequate or small for gestational age, mode of delivery, multiple birth, duration of hospital stay, number of days on mechanical ventilation) were recorded. Gestational age was based on the last menstrual period and early ultrasound examination; infants with birth weight ≥ 10th percentile or < 10th percentile for gestational age, according to the Fenton Growth Chart [21], were classified respectively as adequate or small for gestational age (AGA/SGA). The occurrence of sepsis, necrotizing enterocolitis (NEC) of stage 2 or higher (according to the classification of Bell et al. [22]), intraventricular haemorrhage (IVH) grade 3 or higher, periventricular leukomalacia (PVL) of grade 2 or higher, retinopathy of prematurity (ROP) of stage 3 or higher and bronchopulmonary dysplasia (BPD) were also collected prospectively. Sepsis was defined by the presence of positive blood and/or cerebrospinal fluid culture. IVH and PVL were detected by brain magnetic resonance imaging examination at 40 weeks postmenstrual age. BPD was defined as treatment with supplemental oxygen at 36 weeks gestation. Corrected age was calculated up to 24 months of life, from the chronological age adjusting for gestational age. Mothers’ nationality and education were also recorded. Mothers’ educational level was used as a measure of socioeconomic status and classified using a 3-point scale, where 1 indicates primary or intermediate school education (≤8 years), 2 indicates secondary school education (9–13 years) and 3 indicates a university degree (>13 years).

### Instruments

#### Bayley scales

The Bayley Scales of Infant Development, 2nd Edition [7] yields two single age-standardized composite scores (range 50–150): a Mental Development Index (MDI), which measures cognition through sensory perception, knowledge, memory, problem solving and early language abilities, and a Psychomotor Development Index (PDI), which assesses fine and gross motor skills.

The third revision of the scales (Bayley Scales of Infant and Toddler Development, 3rd Edition) [8] produces three composite scores: the Cognitive scale (range 55–145), which assesses sensorimotor development, exploration and manipulation, object relatedness, concept formation, memory and simple problem solving; the Language scale (range 45–155), which consists of Receptive Communication (verbal comprehension, vocabulary) and Expressive Communication (babbling, gesturing and utterances) subtests; and the Motor scale (range 45–155), which consists of Fine Motor (grasping, perceptual-motor integration, motor planning and speed) and Gross Motor (sitting, standing, locomotion and balance) subtests.

Both editions of the Bayley Scales have index mean scores of 100 (SD ± 15). In the present study, an index composite score of < 70 (>2 SD below the mean) is defined to indicate severe impairment, while an index composite score of 70–84 (>1 SD below the mean) is defined to indicate mild impairment. Index composite scores ≥ 85 are defined here to indicate normal development.

Because neither the Bayley-II nor the Bayley-III has been normed in Italy, the USA norms of the scales were used in this study [7, 8]. The Bayley-II administration manual was translated into Italian through the back-translation method. Before starting the study, the Italian version of the Bayley-II administration manual was tested with a group of infants to clarify any doubts on item comprehension. For the Bayley-III, the Italian validated translation of the administration manual was used [23].

#### Griffiths mental development scales revised

The Griffiths Mental Development Scales Revised (Griffiths) assess the development of infants from birth to 24 months [16]. They comprise five subscales (range 50–150): Locomotor, Personal-Social, Hearing and Speech, Eye and Hand Coordination and Performance. The subscales yield standardized scores for each domain (mean 100, SD 16) and a composite General Quotient (mean 100, SD 12).

For each subscale, a standardized score < 68 (>2 SD below the mean) indicates severe impairment, and a standardized score 68–83 (>1 SD below the mean) indicates mild impairment. Finally, a standardized score ≥ 84 indicates normal development.

As for the General Quotient, severe impairment is defined in the present study to be indicated by a standardized score < 76 (>2 SD below the mean), while mild impairment is categorised here with a standardized score 76–87 (>1 SD below the mean). A standardized score ≥ 88 is defined to indicate normal development.

Because normative data of the Griffiths Mental Development Scales Revised are not available in our country, we referred to the 1996 UK norms. The Manual of the Griffiths Mental Development Scales Revised was translated into Italian through the back-translation method. Before starting the study, the Italian version of the Griffiths Mental Development Scales Revised Manual was tested with a group of infants to clarify any doubts on item comprehension. Since 2007, the Italian-validated translation of the administration manual has been used [24].

### Procedure

Infants underwent evaluation of the neurodevelopmental outcome at 24 months corrected age. Each infant was assessed by two trained and licensed examiners (one administering the Griffiths and the other the Bayley Scales in different sessions on the same day), both blind to the child’s performance on the other test. Infants born between 2003 and 2006 (Group 1) were assessed using Griffiths and Bayley-II, while infants born between 2007 and 2010 (Group 2) were assessed with Griffiths and Bayley-III. Infants were randomly first administered either the Griffiths or the Bayley Scales to avoid a possible test order effect. A short break of 30 min was planned between the two tests to allow the infant to rest and adjust for fatigue. Except for the edition of the Bayley Scales administered, the two groups underwent the same follow-up assessment procedures.

According to Vohr [10], children who could not be assessed because they were too severely impaired (*n* = 4 Quadriplegic Cerebral Palsy) were assigned scores as follows: 49 in the Bayley-II MDI and PDI, 54 in the Bayley-III Cognitive scale, 44 in the Bayley-III Language and Motor scales and 49 in the Griffiths GQ and sub-quotients.

### Statistical analyses

The homogeneity between the two groups of infants has been verified using a confidence interval of 95 % for the differences between the investigated variables expressed as mean or percentage. To evaluate if any infant (sex, gestational age, birth weight below the 10th percentile, being a twin, having siblings, oxygen dependency at 36 weeks postmenstrual age, magnetic resonance imaging, ROP, need for mechanical ventilation) and/or maternal variable (education, age and nationality) were associated with belonging or not to one of the two study groups, a multivariate logistic regression model was performed.

A first comparison between the results obtained at 24 months corrected age by the Bayley and the Griffiths scales was done by comparing the mean values and the 95 % confidence intervals. The obtained scores were then classified as mildly impaired (Bayley Composite Scores or Griffiths Quotients > 1 SD below the mean) or severely impaired (Bayley Composite Scores or Griffiths Quotients > 2 SD below the mean), in accordance with other authors [4, 10, 25]. Concordance between the results given by the different scales was measured using weighted K Cohen and considered poor, fair, good or excellent with Cohen’s kappa 0–0.4, 0.4–0.6, 0.6–0.8, > 0.8, respectively [26]. Taking the results obtained at 24 months corrected age with the Griffiths as the gold standard, steps were taken to calculate the sensitivity, specificity and Youden’s index for the two Bayley editions. The Youden’s Index (sensitivity + specificity-1), with values between 0 and 1, measures the maximum potential effectiveness of a screening test.

**Fig. 2:**
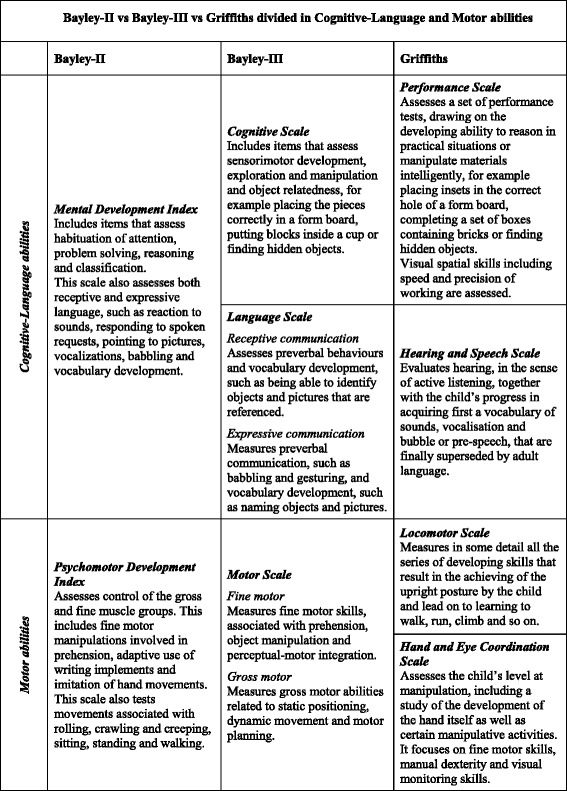
Bayley-II vs Bayley-III vs Griffiths divided into Cognitive language and motor abilities. Manual definitions of Bayley and Griffiths Subscales, grouped in comparable domains: Cognitive language and motor abilities

As noted before, Bayley-II MDI includes both cognitive and language abilities, while both the Bayley-III and the Griffiths Scales yield separate scores (Cognitive and Language vs Hearing and Speech and Performance respectively). The same issue was raised for fine and gross motor abilities, measured together by the Bayley-II PDI and Bayley-III Motor Scale and separately by the Griffiths Scales (Locomotor and Eye and Hand Coordination Scales). Therefore, to compare the Bayley and Griffiths results, subscales that measured the same dimensions, as inferred by the manuals, were grouped together [Fig. 2] as follows, to have homogeneous and comparable domains:

- Griffiths Hearing and Speech-Performance Quotients (mean) vs Bayley-II MDI and vs Bayley-III Cognitive-Language Composite Scores (mean)
- Griffiths Locomotor-Eye and Hand Coordination Quotients (mean) vs Bayley-II PDI and vs Bayley-III Motor Composite Score

## Results

Maternal and infants’ basic characteristics are shown in Table 1.

**Table 1:** Maternal and infant characteristics

Characteristics	Group 1 (*n* = 92)	Group 2 (*n* = 102)	C.I. 95 % of differences
Maternal			
Age, years (mean)	34.2	34.4	−1.22–1.65
University degree, %	23.9	33.3	−4.2–23.1
Non-Italian nationality %	19.6	19.6	−12.2–12.2
Infant			
Birth weight, g, (mean)	796.0	813.3	−18.2–49.4
GA, weeks, (mean)	27.7	27.2	−0.1–1.1
Males, %	43.5	44.1	−14.4–15.6
SGA, %	50.0	38.2	−3.1–2.74
Multiple birth, %	18.5	38.2	6.3–33.16
Cesarean delivery, %	92.4	92.2	−8.3–8.7
Sepsis, %	37.0	27.5	−4.7–23.7
NEC stage 2–3, %	2.2	4.9	−3.5–8.9
IVH grade 3–4, %	2.2	5.9	−2.8–10.2
PVL, %	1.1	2.0	−3.6–5.4
BPD, %	43.4	35.3	−6.6–22.9
ROP grade 3–4, %	16.3	14.7	−9.6–12.8
Days in hospital, (mean)	95.2	104.2	−3.7–21.6
Days on ventilation, (mean)	14.3	12.4	−2.8–6.5

The mean age at testing was 23.0 months (SD 1.7 months; range 22 months and 16 days-24 months and 15 days) of corrected age. Although 19.6 % of mothers in both groups were not Italian, all infants attended a kindergarten or a preschool education programme and so were exposed to Italian as a primary language in their community environment.

As shown in Table 1, there were no significant differences between the two groups for each of the variables considered, with the exception of a much higher percentage of multiple pregnancies in the second group.

The logistic regression model showed that the two study groups were homogenous with regard to maternal and infants’ characteristics (likelihood ratio 21:36, df = 16, *p* = 0.1650 and rsquare rescaled = 0.1560).

Table 2 shows the means (95 % CI) of the Griffiths Hearing and Speech-Performance vs Bayley-II MDI or vs Bayley-III Cognitive-Language and the Griffiths Locomotor-Eye and Hand Coordination (mean) vs Bayley-II PDI or vs Bayley-III Motor composite scores.

**Table 2:** Griffiths vs Bayley-II – Bayley-III

	Mean (C.I. 95 %)	Mean (C.I. 95 %)
Group 1	Griffiths	Bayley-II
Cognitive-Language abilities^a^	86.0 (82.0–89.9)	79.4 (74.7–84.0)
Motor abilities^b^	91.7 (87.9–95.5)	83.8 (79.6–87.9)
Group 2	Griffiths	Bayley-III
Cognitive-Language abilities^c^	90.3 (87.2–93.5)	90.2 (87.6–92.8)
Motor abilities^d^	91.8 (88.4–95.2)	93.0 (89.6–96.4)

^a^Griffiths Hearing and Speech-Performance Quotients (mean) vs Bayley-II MDI

^b^Griffiths Locomotor-Eye and Hand Coordination Quotients (mean) vs Bayley-II PDI

^c^Griffiths Hearing and Speech-Performance Quotients (mean) vs Bayley-III Cognitive-Language Composite Scores (mean)

^d^Griffiths Locomotor-Eye and Hand Coordination Quotients (mean) vs Bayley-III Motor Composite Score

The Bayley-II MDI composite score was 6.6 points lower than the Griffiths Hearing and Speech-Performance combined score, whereas the Bayley-III Cognitive-Language combined score was almost equal to it.

For the Griffiths Locomotor-Eye and Hand Coordination combined score, the discrepancy with the Bayley-II PDI composite score was even larger (7.9 points lower), whereas the Bayley-III Motor composite score was only 1.2 points higher. Table 3 reports the concordance between Griffiths and Bayley II/Bayley III.

**Table 3:** Concordance between Griffiths and Bayley-II (Group 1) or Bayley-III (Group 2)

	Concordance (%)	Weighted K	C.I. 95 % of K
Group 1			
Cognitive-Language abilities^a^	70.7	0.63	0.51–0.75
Motor abilities^b^	67.4	0.50	0.35–0.65
Group 2			
Cognitive-Language abilities^c^	89.2	0.80	0.69–0.92
Motor abilities^d^	90.2	0.81	0.69–0.93

^a^Griffiths Hearing and Speech-Performance Quotients (mean) vs Bayley-II MDI

^b^Griffiths Locomotor-Eye and Hand Coordination Quotients (mean) vs Bayley-II PDI

^c^Griffiths Hearing and Speech-Performance Quotients (mean) vs Bayley-III Cognitive-Language Composite Scores (mean)

^d^Griffiths Locomotor-Eye and Hand Coordination Quotients (mean) vs Bayley-III Motor Composite Score

Griffiths and Bayley-III composite scores for both cognitive-language and motor abilities showed an excellent concordance. On the contrary, concordance between Griffiths and Bayley-II was lower, especially with regard to motor skills. Table 4 outlines the ranges of developmental impairment. Compared with the Griffiths, the Bayley-II showed consistently higher rates of severe impairment both in cognitive and language abilities (14.1 % more infants) and in motor skills (15.3 % more infants). There was a higher agreement between the Bayley-III and the Griffiths rates with regard to mild and severe impairment in all domains, except for motor mild impairment, which appeared to occur in a slightly lower percentage of infants when the Bayley-III was used (7.8 % fewer infants). The comparison between single subscales revealed that the Bayley-III Cognitive Index detected 7.9 % fewer infants as being mildly impaired and 4.9 % fewer infants as being severely impaired compared with the Griffiths Performance subscale. The Bayley-III Language Index showed mild impairment in a higher percentage of cases (4.9 % more infants) and severe impairment in a lower percentage of cases (4.9 % fewer infants) compared with the Griffiths Hearing and Speech subscale.

**Table 4:** Rates of developmental impairment

	*n* (%)	*n* (%)	
Group 1	Bayley-II	Griffiths	
Cognitive-Language abilities^a^ within normal limits	40 (43.5)	54 (58.7)	
Cognitive-Language abilities^a^ mild impairment	21 (22.8)	20 (21.7)	
Cognitive-Language abilities^a^ severe impairment	31 (33.7)	18 (19.6)	
Motor abilities^b^ within normal limits	53 (57.6)	66 (71.7)	
Motor abilities^b^ mild impairment	13 (14.1)	14 (15.2)	
Motor abilities^b^ severe impairment	26 (28.3)	12 (13.0)	
Group 2	Bayley-III	Griffiths	
Cognitive-Language abilities^c^ within normal limits	78 (76.5)	74 (72.6)	
Cognitive-Language abilities^c^ mild impairment	16 (15.7)	17 (16.7)	
Cognitive-Language abilities^c^ severe impairment	8 (7.8)	11 (10.8)	
Motor abilities^d^ within normal limits	84 (82.4)	77 (75.5)	
Motor abilities^d^ mild impairment	7 (6.9)	15 (14.7)	
Motor abilities^d^ severe impairment	11 (10.8)	10 (9.8)	
	*n* (%)	*n* (%)	*n* (%)
	Bayley-III	Griffiths	
Group 2-for single subscales			
Cognitive abilities^e^ within normal limits	87 (85.3)	74 (72.5)	
Cognitive abilities^e^ mild impairment	8 (7.8)	16 (15.7)	
Cognitive abilities^e^ severe impairment	7 (6.9)	12 (11.8)	
Language abilities^f^ within normal limits	75 (73.5)	75 (73.5)	
Language abilities^f^ mild impairment	17 (16.7)	12 (11.8)	
Language abilities^f^ severe impairment	10 (9.8)	15 (14.7)	
Motor abilities^g^ within normal limits	84 (82.4)	73 (71.6)	84 (82.4)
Motor abilities^g^ mild impairment	7 (6.9)	8 (7.8)	9 (8.8)
Motor abilities^g^ severe impairment	11 (10.8)	21 (20.6)	9 (8.8)

^a^Bayley-II MDI vs Griffiths Hearing and Speech-Performance Quotients (mean)

^b^Bayley-II PDI vs Griffiths Locomotor-Eye and Hand Coordination Quotients (mean)

^c^Bayley-III Cognitive-Language Composite Scores (mean) vs Griffiths Hearing and Speech-Performance Quotients (mean)

^d^Bayley-III Motor Composite Score vs Griffiths Locomotor-Eye and Hand Coordination Quotients (mean)

^e^Bayley-III Cognitive Composite Score vs Griffiths Performance Quotient

^f^Bayley-III Language Composite Score vs Griffiths Hearing and Speech Quotient

^g^Bayley-III Motor Composite Score vs Griffiths Locomotor Quotient vs Eye and Hand Coordination Quotient

Finally, considering motor skills, the Bayley-III Motor Index highly agreed with the Griffiths Eye and Hand Coordination subscale but identified 9.8 % fewer infants as being severely impaired compared with the Griffiths Locomotor subscale.

As noted in Table 5, in comparison to the Griffiths Scales, the sensitivity of the Bayley-II was greater than that of the Bayley-III, especially for cognitive-language abilities. On the contrary, Bayley-III appeared to have an increased specificity compared with its previous edition. However, the Youden’s Index (combining sensitivity and specificity) reveals much higher values for the Bayley-III than for the Bayley-II both for cognitive language and motor abilities.

**Table 5:** Sensitivity, specificity and Youden’s Index of Bayley-II and Bayley-III vs Griffiths

	Sensitivity	Specificity	Youden’s index
	(%)	(%)	(%)
Group 1			
Cognitive-Language abilities^a^	97.4	72.2	69.6
Motor abilities^b^	80.8	72.7	53.5
Group 2			
Cognitive-Language abilities^c^	78.6	97.3	75.9
Motor abilities^d^	68.0	98.7	66.7

^a^Bayley-II MDI vs Griffiths Hearing and Speech-Performance Quotients (mean)

^b^Bayley-II PDI vs Griffiths Locomotor-Eye and Hand Coordination Quotients (mean)

^c^Bayley-III Cognitive-Language Composite Scores (mean) vs Griffiths Hearing and Speech-Performance Quotients (mean)

^d^Bayley-III Motor Composite Score vs Griffiths Locomotor-Eye and Hand Coordination Quotients (mean)

## Discussion

Our study shows that the Bayley-II and the Bayley-III yield significantly different outcomes, with the latter displaying higher composite scores both in the cognitive-language and motor abilities. Concerning the comparison with the Griffiths Scales, the Bayley-III mean composite scores revealed a higher agreement than the previous edition.

The increased scores obtained using the Bayley-III, compared with the previous edition, might be because of the improved outcomes of ELBW/ELGAN infants over time [27]. However, it must be taken into account that, in our cohort, there were no significant differences between the rates of impairment detected using the Griffiths throughout the whole study period. A possible explanation of our finding could rely on the changes in the structure of the scales. Indeed, in the Bayley-III, Cognitive and Language scores are separated so as to minimize the effects of language impairment on cognitive assessment. Thus, it can be speculated that the MDI scores were lower because cognitive assessment was negatively affected by the presence of impairments in language abilities. In addition, the Bayley-II uses item sets with established start and stop points, which may create an artificial ceiling. On the contrary, in the Bayley-III, although a start point based on age is also present, the examiner continues to administer the test items until the child receives scores of 0 for five consecutive items. Consequently, a bright child is allowed to achieve a higher level. Furthermore the Griffiths basal and ceiling rules are similar to those of the Bayley-III, as the manual recommends that the child successfully answers six consecutive items for each subscale, while administration should be discontinued when the child misses six consecutive items. It is therefore clear that both the test design and the administration rules of Bayley-III are more consistent with the Griffiths, which may explain the higher agreement between the scales’ outcomes. However, concern persists that the Bayley-III may tend to underestimate both mild and severe neurodevelopmental impairment.

Indeed, whereas the degree of concordance between the Griffiths and the Bayley-III is high at an overall (non-severity-specific) level, a more detailed analysis on single subscales shows that the Bayley-III detects 5 % fewer infants as being severely impaired in language abilities and 13 % fewer infants as being mildly and severely impaired in cognitive abilities.

Our findings suggest that scores classified as “severe impairment” and “mild impairment” according to the Griffiths tend to shift up towards “mild impairment” and “normal” levels, respectively, when using the Bayley-III.

It is possible that the Bayley-III identifies fewer infants with language impairment because it separates the receptive and expressive subscales, so a child can reach a higher score by passing all the receptive items even if the production is compromised. On the contrary, as the Griffiths Hearing and Speech subscale mixes production and comprehension items, the achievement of a high score requires a greater integration of verbal skills. We also hypothesize that the Griffiths Performance subscale requires a greater integration of cognitive functions, providing a score that is more consistent with the actual level of the infant’s cognitive functioning. Conversely, the Bayley-III Cognitive Index consists of a greater number of items with simpler and more graded tasks, so it is easier for a child to gain a higher score. The Bayley-III combination of fine and gross motor abilities makes it difficult to identify specific impairments in one of the two areas. Indeed, the comparison with the Griffiths Locomotor and Eye and Hand Coordination subscales shows that the Bayley-III Motor Index fails in identifying 10 % of severe gross motor impairments.

Our findings on the Bayley-II and the Bayley-III outcomes are consistent with previous studies reporting > 7 points of difference between the Bayley-II MDI and the Bayley-III Cognitive score [28].

In cohorts of infants born earlier than 25 weeks’ gestation, Hintz et al. [29], using the Bayley-II at 18–22 months’ corrected age, reported rates of mild to severe cognitive impairment ranging from 40 to 47 %, while mild to severe motor impairment ranged from 31 to 32 %. In our cohort, the rates of mild and severe developmental impairment, according to the Bayley-II, were slightly lower than those commonly reported in the literature. This is probably because of the higher assessment age of our study group (24 months corrected age) that may have reduced the impact of health and medical issues on child neurodevelopmental outcome. On the contrary, the rates of mild and severe impairment found in the present study according to the Bayley-III slightly exceeded those reported by Anderson et al. [30], who found mild to severe cognitive impairment in 10 and 3 %, respectively, and mild to severe language impairment in 16 % of their preterm cohort.

As for the Griffiths outcomes, Claas et al. [25], studying a cohort of preterm infants with birth weight ≤ 750 g at 2 years, reported that none of the infants assessed with the Griffiths had a GQ of < 76 (<2 SD), whereas 9.6 % infants assessed with the Bayley-II had a MDI < 70. Similarly, in our cohort, rates of severely impaired infants according to the Griffiths (ranging from 10 to 20 %) were found to be lower than those revealed by the Bayley-II (ranging from 28 to 34 %), but greater than those of the Bayley-III (ranging from 8 to 11 %).

Our rates of agreement between the Griffiths and the Bayley-III average scores are higher than those reported by Milne et al. [31] Y. The authors, comparing a cohort of 100 preschoolers referred for assessment of developmental impairment at 32 months using the Bayley-III and reassessed at 52 months using the Griffiths Scales, found that the Bayley-III average composite scores identify 7 % fewer children as being mildly impaired and 28 % fewer children as being severely impaired compared with the Griffiths General Quotient. Thus, underestimation of the Bayley-III, in comparison to the Griffiths Scales, seems more evident at later ages even though it must be taken into account that 59 % of children studied by Milne et al. were affected by autism.

The main strength of our study is that it provides a comparison with one of the most recognized instruments for neurodevelopmental assessment, the Griffiths, which gives a standardized independent criterion on which performances at the Bayley Scales can be referred. The main limitation of the current study is that the two editions of the Bayley Scales were not administered to the same study group. In addition, because none of the neurodevelopmental assessments used in the present study have been normed in Italy, we had to use the USA norms for the Bayley-II and the Bayley-III and the UK norms for the Griffiths.

## Conclusions

The findings of our study indicate that the Bayley-III has a higher agreement with the Griffiths Scales compared with the Bayley-II. Conversely, the Bayley-II yields higher rates of severe impairment than the Griffiths both in cognitive-language and motor abilities.

However, it is clinically relevant to note that the Bayley-III slightly tends to shift up scores classified as “severe impairment” and “mild impairment” according to the Griffiths towards “mild impairment” and “normal range”, thus making it sometimes difficult to ascertain the real extent of neurodevelopmental impairment.

These findings have important implications for clinical services, follow-up programmes and clinical trials that rely on the Bayley-III for the assessment of developmental impairment. As the Bayley scores are often used to determine eligibility for early intervention services, the use of the Bayley-III may result in the lack of qualification for early intervention programmes of infants that would have been previously eligible. On the basis of the present findings, the use of multiple measures could be recommended to assess neurodevelopmental outcome of ELBW infants at the age of 2 years. Additional studies are needed to replicate the current findings in larger populations and at different ages of assessment.

## Abbreviations

ELBW: Extremely low birth weight
ELGAN: Extremely low gestational age newborns
AGA/SGA: Adequate/small for gestational age
NEC: Necrotizing enterocolitis
IVH: Intraventricular hemorrhage
PVL: Periventricular leukomalacia
ROP: Retinopathy of prematurity
BPD: Bronchopulmonary dysplasia
MDI: Mental development index
PDI: Psychomotor development index
